# Disulfidptosis classification of pancreatic carcinoma reveals correlation with clinical prognosis and immune profile

**DOI:** 10.1186/s41065-025-00381-z

**Published:** 2025-02-22

**Authors:** Jiangmin Shi, Liang Zhao, Kai Wang, Jieqiong Lin, Jianwei Shen

**Affiliations:** Department of Gastroenterology, Ningbo Medical Center Lihuili Hospital (Lihuili Hospital Affiliated to, Ningbo University), Ningbo, Zhejiang Province 315040 P.R. China

**Keywords:** Disulfidptosis, Long noncoding RNA (lncRNA), Pancreatic carcinoma, Prognostic signature, Immune microenvironment, Drug sensitivity

## Abstract

**Background:**

Disulfidptosis, a novel form of metabolism-related regulated cell death, is a promising intervention for cancer therapeutic intervention. Although aberrant expression of long‐chain noncoding RNAs (lncRNAs) expression has been associated with pancreatic carcinoma (PC) development, the biological properties and prognostic potential of disulfidptosis-related lncRNAs (DRLs) remain unclear.

**Methods:**

We obtained RNA-seq data, clinical data, and genomic mutations of PC from the TCGA database, and then determined DRLs. We developed a risk score model and analyzed the role of risk score in the predictive ability, immune cell infiltration, immunotherapy response, and drug sensitivity.

**Results:**

We finally established a prognostic model including three DRLs (AP005233.2, FAM83A-AS1, and TRAF3IP2-AS1). According to Kaplan–Meier curve analysis, the survival time of patients in the low-risk group was significantly longer than that in the high-risk group. Based on enrichment analysis, significant associations between metabolic processes and differentially expressed genes were assessed in two risk groups. In addition, we observed significant differences in the tumor immune microenvironment landscape. Tumor Immune Dysfunction and Rejection (TIDE) analysis showed no statistically significant likelihood of immune evasion in both risk groups. Patients exhibiting both high risk and high tumor mutation burden (TMB) had the poorest survival times, while those falling into the low risk and low TMB categories showed the best prognosis. Moreover, the risk group identified by the 3-DRLs profile showed significant drug sensitivity.

**Conclusions:**

Our proposed 3-DRLs-based feature could serve as a promising tool for predicting the prognosis, immune landscape, and treatment response of PC patients, thus facilitating optimal clinical decision-making.

**Supplementary Information:**

The online version contains supplementary material available at 10.1186/s41065-025-00381-z.

## Introduction

Pancreatic carcinoma (PC) is a common malignant tumor that has increased in incidence over the past few years, accounting for approximately 2% of all cancers and causing 5% of cancer-related deaths [1]. PC is one of the most aggressive and chemo-resistant cancers, mainly due to the diversity of genetic mutations leading to a highly heterogeneous disease. Based on that early surgical resection is currently the only effective treatment, for PC patients, early diagnosis and timely surgical intervention are urgent. However, most patients have no obvious symptoms as the disease develops and progresses to advanced stages. Traditional clinical predictive factors such as tumor stage (T), lymph node involvement (N), and distant metastasis (M) are widely used to predict prognosis and aid in the treatment [2]. However, with advances in molecular biology and high-throughput technologies, it is critical to develop reliable and effective predictive biomarkers to identify unique subgroups of PC patients. These biomarkers will become indispensable tools to guide personalized and favorable treatment strategies.

Accidental cell death (ACD) and regulatory cell death (RCD) are common types of cell death [3]. In recent years, many emerging models of RCD have attracted great attention and a new mode of cell death was identified by Liu named disulfidptosis [4]. Most cancer cells primarily obtain cysteine by uptake of extracellular cysteine (an oxidized cysteine dimer) through the solute carrier family 7 member 11 (SLC7A11) [5–7]. After entering the cell, each cysteine is reduced to two molecules of cysteine in an NADPH dependent reaction, and subsequently cysteine is used for the biosynthesis of glutathione and other metabolic processes, such as protein synthesis [8]. SLC7A11 has a recognized role in maintaining intracellular glutathione levels and protecting cells from oxidative stress-induced cell death (such as ferroptosis) [9–11], and SLC7A11 is often overexpressed in cancer [12–14], which can protect them from ferroptosis, but Gan et al. found that it also has vulnerabilities [15]. Cysteine contains active disulfide bonds and can react with various proteins throughout the cell, exhibiting toxic effects. Cells use the reducing agent NADPH to neutralize cysteine, while NAPDH is mainly provided by glucose. Therefore, cells overexpressing SLC7A11 are addicted to glucose, and a lack of this energy source can lead to toxic accumulation of cysteine and trigger disulfide death [4]. Gan’s research team found that the accumulation of cysteine leads to the accumulation of disulfide bonds in the actin cytoskeleton, ultimately resulting in cell death [15]. Inhibitors of cell apoptosis, ferroptosis, and other cell death mechanisms have no effect on this process, indicating that disulfide mediated cell death is a unique pathway. Disulfidptosis provides a new pathway for destroying tumor cells that develop resistance to ferroptosis, while minimizing collateral toxicity to the greatest extent possible. Moreover, this study has shown that disulfidptosis also has the ability to affect immune infiltration, suggesting the occurrence of disulfidptosis may provide a new approach for tumor treatment.

Several recent studies have constructed predictive disulfidptosis-related genes (DRGs) or disulfidptosis-related lncRNAs (DRLs) models, which were stable and reliable for predicting cancer prognosis [16, 17]. In this study, a reliable marker for DRLs was established for predicting prognosis and guiding clinical treatment. Survival time prediction, biological properties, immune infiltration, tumor mutational burden (TMB), and drug responsiveness were investigated using a predictive model of three DRLs. Our findings are expected to provide new perspectives and approaches for therapy strategies of PC patients.

## Materials and methods

### Data acquisition and processing

Consolidated transcriptome expression matrix and clinical data of PC patients were obtained from the Cancer Genome Atlas (TCGA) database (accessed on 11 September 2023)), which included 179 tumor specimens and 4 normal samples.

### Identification the expression matrix of disulfidptosis-related genes

A total of 24 DRGs, including FLNA, FLNB, MYH9, TLN1, ACTB, MYL6, MYH10, CAPZB, DSTN, IQGAP1, ACTN4, PDLIM1, CD2AP, INF2, SLC7A11, SLC3A2, NUBPL, NDUFS1, GYS1, OXSM, LRPPRC, NDUFA11, NCKAP1, and RPN1, were summarized in a related review [4]. The DRGs and DRLs expression matrix was retrieved and utilized to draw the Sankey diagram.

### Construction and validation of prognostic features

PC patients were randomized in a 1:1 ratio into training and testing groups [18]. DLRs resulted from univariate Cox regression were kept for the subsequent stage. In addition, a total of three prognostic DRLs were obtained by the least absolute shrinkage and selection operator (LASSO) and multivariate Cox regression analysis. Afterwards, we developed the prognostic model utilizing the three DRLs. Based on the median risk score, patients in the training group, test group, and all group were categorized into low- and high-risk groups respectively. Overall survival (OS) was predicted by Kaplan-Meyer (K-M) survival analysis for the high-risk and low-risk groups. Moreover, the model’s accuracy was assessed using the receiver operating characteristic curve (ROC), nomograms, and calibration curves.

### Analysis of functional enrichment

To explore potential biological functions among clusters, we performed gene set variation analysis. Functional enrichment requirements were derived from Molecular Signatures Database (MSigDB) data. The “GSVA” package was used to identify genomic enrichment pathways. Gene Ontology (GO) and the Kyoto Encyclopedia of Genes and Genomes (KEGG) and Gene Set Enrichment Analysis (GSEA) analysis were performed [19].

### Analysis of tumor microenvironment and immune infiltration

To investigate the correlation between the subtypes identified by clustering and the presence of tumor microenvironment (TME), we used an estimation method to the scores of all samples. We derived TME scores, matrix scores, and immune scores for all PC patients. We used GSEA to assess differential immune profiles in clustering. We used “GSEABase” and “GSVA” for immune assessment. We analyzed immune-related genes between clusters and plotted the results as box plots. Tumor Immune Dysfunction and Exclusion (TIDE) algorithm was used to assess the potential efficacy of tumor immunotherapy.

### Analysis of tumor mutation and drug sensitivity

Tumor mutation burden (TMB) generates new immunogenicity and is thought to predict immune checkpoint blockade response [20]. We mapped the mutation profiles of the two risk groups to visualize the frequency and type of mutated genes and used violin plots to visualize the differences between the TMB risk groups. Expression data and sensitivity data for targeted drugs were obtained from Genetics of Drug Sensitivity in Cancer (GDSC). We analyzed the differences in the sensitivity of the two risk groups to different therapeutic drugs.

### Statistical analysis

Correlation analysis was performed with the Pearson and Spearman correlation test. Survival analysis was performed with the K-M plot and compared by the log-rank method. Finally, the univariate and multivariate Cox regression analysis determined the independent prognostic predictors. *p* < 0.05 was set as the cut-off value. All statistical analyses were conducted with R 4.3.1**.**

## Results

### Characterization of DRLs based molecular subgroups in PC

**Fig. 1:**
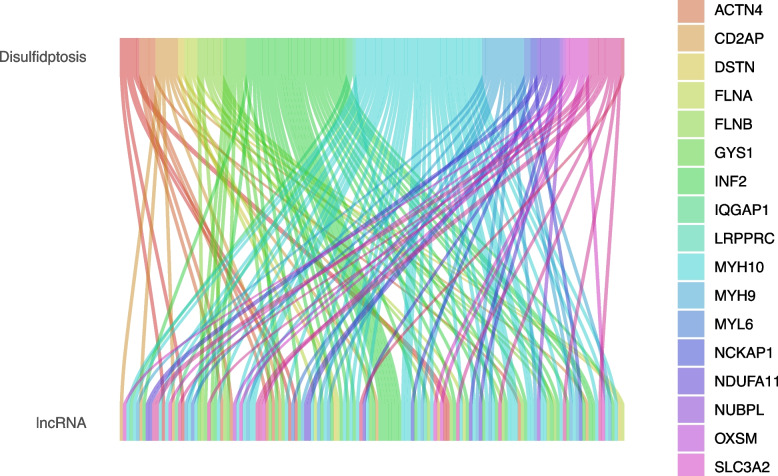
The Sankey relation between DRGs and DRLs

One hundred seventy nine PC patients with comprehensive clinical data from the TCGA database were randomly allocated into two groups. The clinical characteristics of the patients in the two groups were listed in Table 1, which showed no significant differences between the two groups. Twenty-four DRGs identified based on the literature review and previous studies were used to determine DRLs (Fig. 1).

**Table 1:** The clinical characteristics of PC patients in training and testing groups

Covariates	Type	Total	Test cohort	Train cohort	*P*-value
Age	< = 65	94 (52.81%)	45 (50.56%)	49 (55.06%)	0.6524
	> 65	84 (47.19%)	44 (49.44%)	40 (44.94%)	
Gender	FEMALE	80 (44.94%)	40 (44.94%)	40 (44.94%)	1
	MALE	98 (55.06%)	49 (55.06%)	49 (55.06%)	
Grade	G1	31 (17.42%)	17 (19.1%)	14 (15.73%)	0.5962
	G2	95 (53.37%)	50 (56.18%)	45 (50.56%)	
	G3	48 (26.97%)	20 (22.47%)	28 (31.46%)	
	G4	2 (1.12%)	1 (1.12%)	1 (1.12%)	
	unknow	2 (1.12%)	1 (1.12%)	1 (1.12%)	
Stage	Stage I	21 (11.8%)	12 (13.48%)	9 (10.11%)	0.1653
	Stage II	147 (82.58%)	70 (78.65%)	77 (86.52%)	
	Stage III	3 (1.69%)	1 (1.12%)	2 (2.25%)	
	Stage IV	4 (2.25%)	4 (4.49%)	0 (0%)	
	unknow	3 (1.69%)	2 (2.25%)	1 (1.12%)	
T	T1	7 (3.93%)	3 (3.37%)	4 (4.49%)	0.8853
	T2	24 (13.48%)	13 (14.61%)	11 (12.36%)	
	T3	142 (79.78%)	70 (78.65%)	72 (80.9%)	
	T4	3 (1.69%)	1 (1.12%)	2 (2.25%)	
	unknow	2 (1.12%)	2 (2.25%)	0 (0%)	
M	M0	80 (44.94%)	39 (43.82%)	41 (46.07%)	0.1366
	M1	4 (2.25%)	4 (4.49%)	0 (0%)	
	unknow	94 (52.81%)	46 (51.69%)	48 (53.93%)	
N	N0	49 (27.53%)	28 (31.46%)	21 (23.6%)	0.3347
	N1	124 (69.66%)	59 (66.29%)	65 (73.03%)	
	unknow	5 (2.81%)	2 (2.25%)	3 (3.37%)

### Recognition of a prognostic DRLs signature

**Fig. 2:**
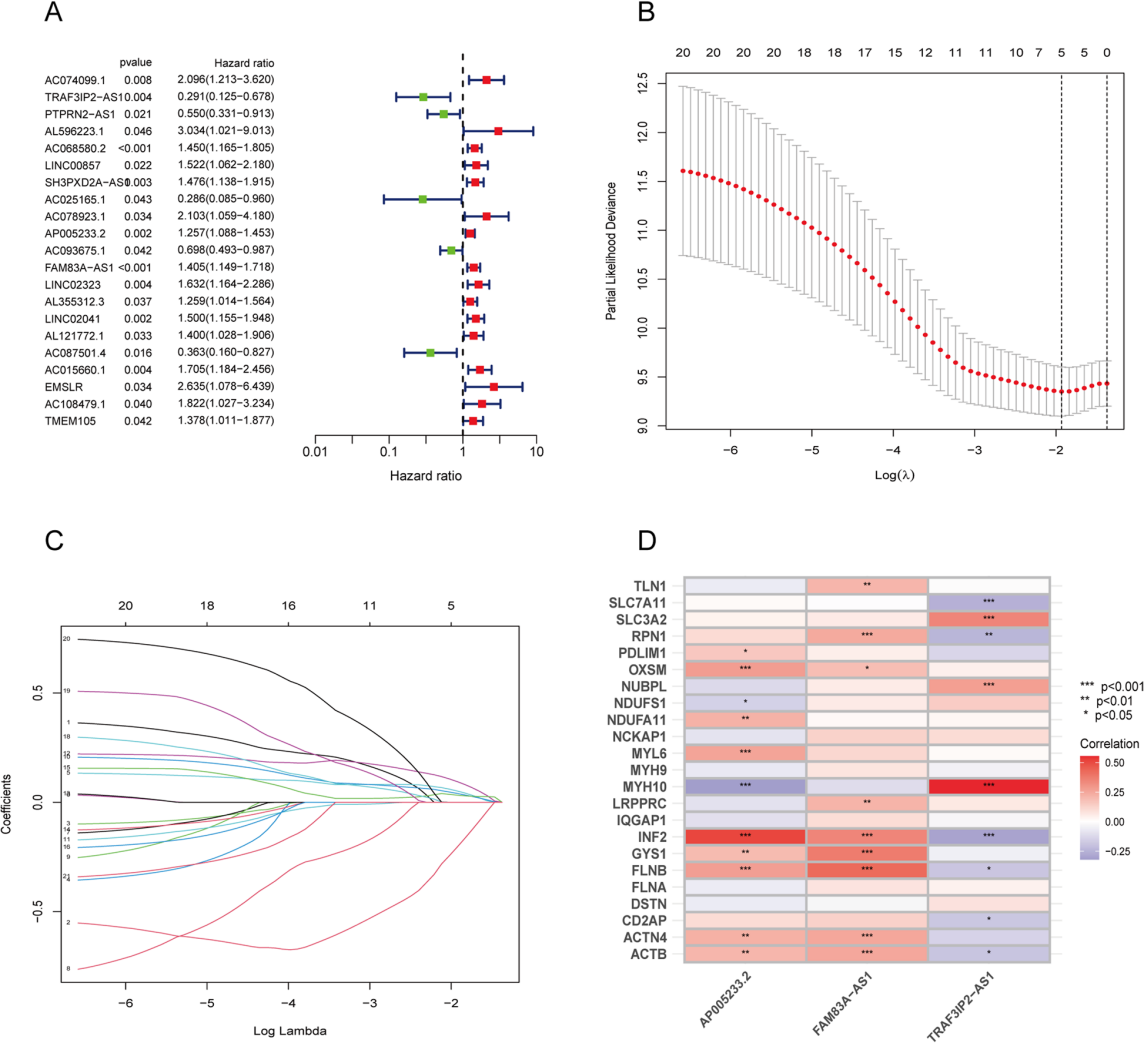
Identification of the prognostic features of pancreatic carcinoma (PC) linked to DRLs. **A** Univariate cox forest map showing the top 21 prognostic DRLs. **B** LASSO coefficient profiles of the expression of DRLs. **C** Selection of the penalty parameter in the LASSO model via tenfold cross-validation. **D** The relationships between the three DRLs and DRGs. *, *p* < 0.05; **, *p* < 0.01; ***, *p* < 0.001

A total of twenty-one DRLs were significantly associated with patients’ OS in the training group by through univariate Cox regression analyses (*p* < 0.05, Fig. 2A). Sixteen DRLs had a hazard ratio (HR) greater than 1, which indicated that they were poor prognostic predictors, whereas the remaining five DRLs were protective factors with HR lower than 1. Furthermore, three candidate lncRNAs were finalized by LASSO and the multivariate Cox regression method (Fig. 2B–C), including AP005233.2, FAM83A − AS1, and TRAF3IP2 − AS1. Additionally, Fig. 2D showed the relationships between the three DRLs and DRGs.

### The risk score could be an independent prognostic factor and assist in predicting clinical outcomes for PC patients

**Fig. 3:**
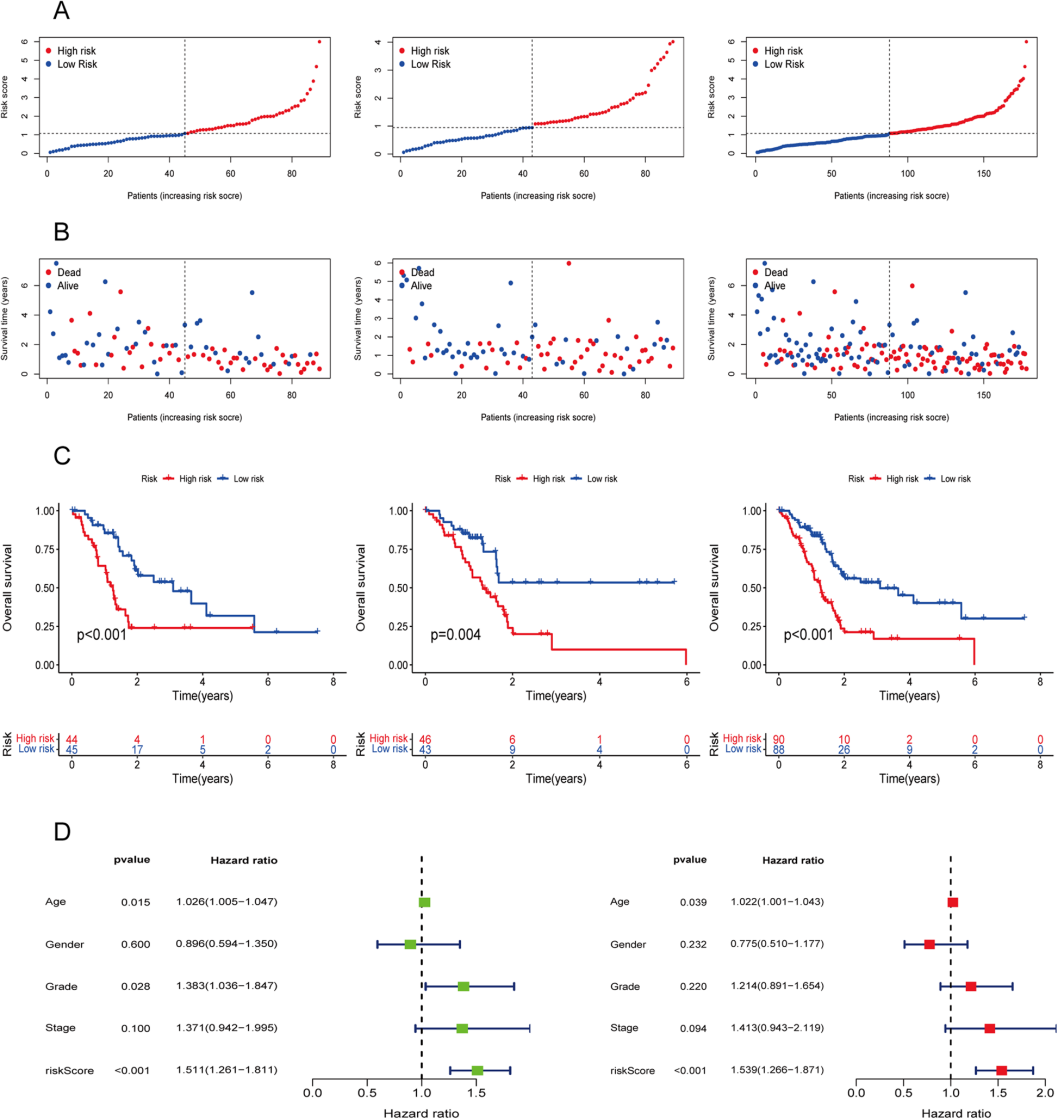
Evaluation and validation of the independent prognostic ability of 3-DRLs signature model in training, testing, and all sets. **A** The distribution of patient with increasing risk scores. **B** The survival time of patients and risk scores. **C** The K-M survival analysis of survival status and overall survival (OS) of PC patients between two risk groups (The red line represents high-risk groups, and the blue line represents low-risk groups). **D** A univariate Cox regression analysis and multivariate Cox regression analysis of clinical variables and risk score

Based on the median risk score for each dataset, patients were categorized into high-risk and low-risk groups for further survival analysis. Figure 3 showed the risk scores and survival of patients in the training, testing and all groups. The results showed a significant correlation between risk scores and patient survival in all datasets. Patients in the high-risk group had significantly lower OS compared to patients with low-risk scores (*p* < 0.05, Fig. 3C). In addition, the mortality rate increased as the risk score increased (Fig. 3A-C). Univariate and multivariate Cox regression analyses showed that the risk group categorized according to the three DRLs features was identified as an independent prognostic factor for PC patients compared to other clinicopathological features (Fig. 3D).

### Validation of the 3-DRLs predictive signature and construction of a nomogram combining clinical characteristics

**Fig. 4:**
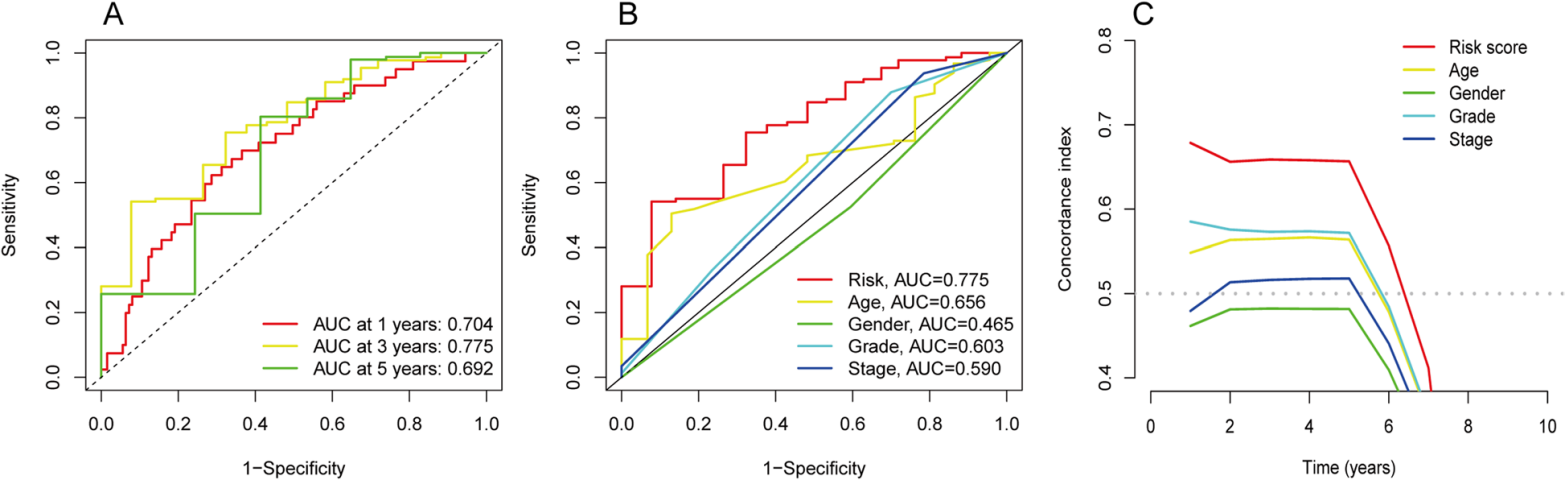
Validation of the predictive model and construction of a nomogram combining clinical characteristics. **A** The ROC curves show the predictive accuracy in 1-, 3-, and 5-year of the predictive risk model. **B** The ROC curves show the predictive accuracy of the predictive risk model and clinicopathological characteristics. **C** ROC curves of the nomogram and clinical features demonstrating superior prediction of prognosis

During the 1-, 3-, and 5-year follow-up periods, the ROC curves demonstrated area under the curve (AUC) values of 0.704, 0.775, and 0.692, respectively (Fig. 4A). Additionally, an ROC curve was generated to verify that the risk score had higher prognostic accuracy compared to other clinical variables such as age, gender, stage, and grade (Fig. 4B). AUC for the risk score was 0.775, indicating strongest predictive capability (Fig. 4B). Moreover, we calculated the C-index through bootstrap resampling and found that the line map based on the 3-DRLs predictive signature had superior accuracy (Fig. 4C).

To improve the utility of the features, we created a predictive nomogram by summing the assigned scores of relevant clinical factors and risk scores on a points scale. This allowed for accurate prediction of the probability of survival. Because of the ultra-short survival of PC patients and limited sample size, we excluded stage III patients and illustrated the selected patient’s probability of 1-, 3-, and 5-years OS in Supplementary Fig. 1. Additionally, we verified the consistency of the nomogram predictions with the actual measured outcome were validated by the calibration curves. As illustrated in Supplementary Fig. 2, the results of the study demonstrated strong agreement between clinical outcomes and predicted values. In conclusion, these results suggest that nomograms combining the predictive and clinical features of 3-DRLs can accurately predict the clinical prognosis of PC patients.

### Prediction of clinical prognosis in patients in high- and low- risk groups

**Fig. 5:**
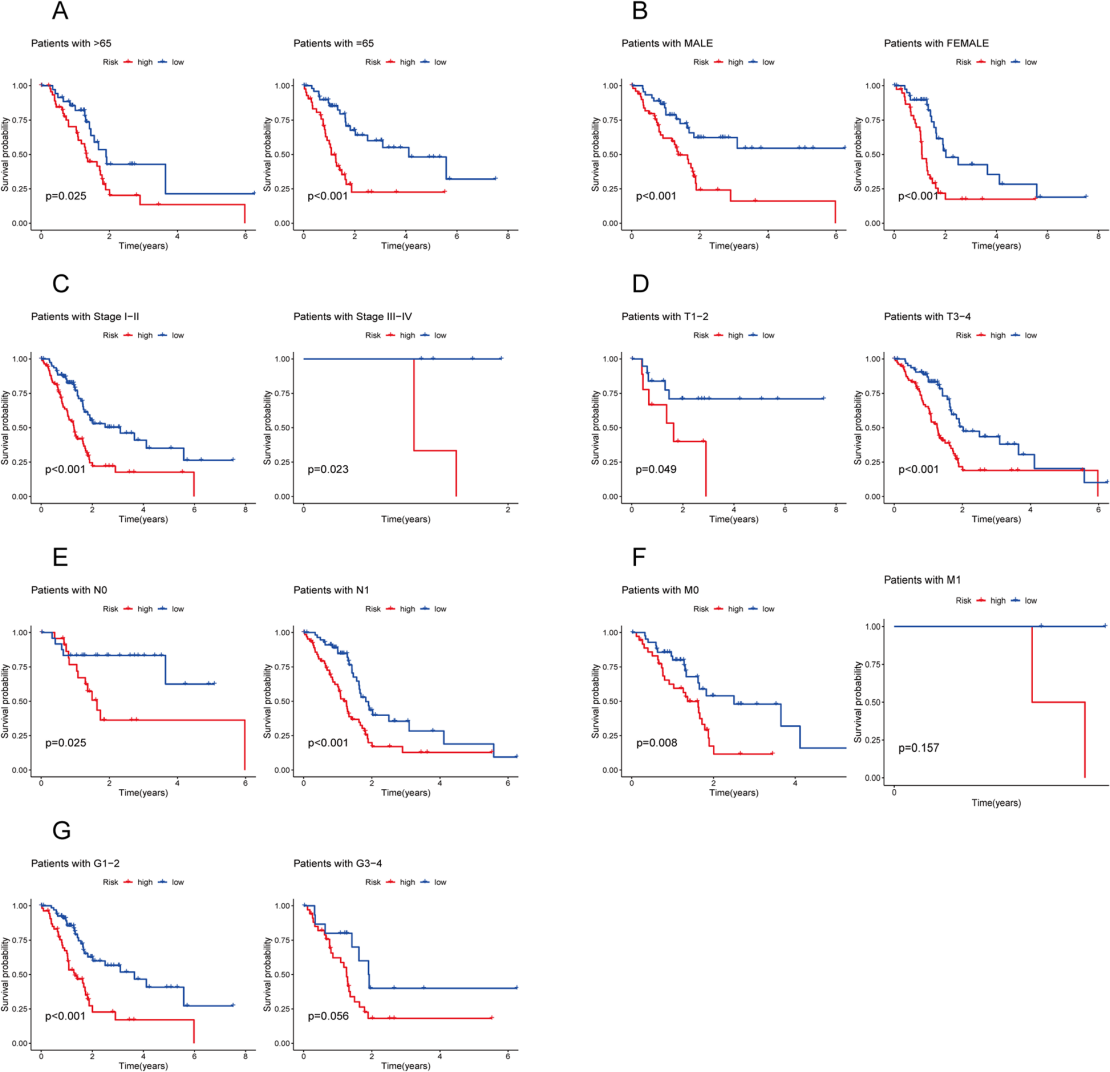
The K—M survival analysis of low- and high-risk patients with different clinical variables. **A** Age (> 65, ≤ 65); **B** Gender (Male, Female); **C** Stage (Stage I-II, Stage III-IV); **D** T stage (T1-2, T3-4); **E** N stage (N0, N1); **F** M stage (M0, M1); **G** Grade (G1-2, G3-4). The red line represents high-risk groups, and the blue line represents low-risk groups

Based on the predictive features of 3-DRLs, we compared the survival rates of PC patients in the high-risk and low-risk groups according to age, gender, stage, and TNM staging (Fig. 5). The results showed that OS was significantly shorter in the high-risk group than in the low-risk group, except for M1 (Fig. 5F) and G3-4 (Fig. 5G). One possible explanation is that the prognosis of advanced PC is poor, and therefore the number of M1 and G3-4 patients is relatively small, which may lead to a certain degree of error in the results. In conclusion, these results suggest that the 3-DRLs prediction model has great potential for predicting PC prognosis and can be applied to a variety of clinical variables.

### Analysis of biological functions by GO, KEGG, and GSEA

**Fig. 6:**
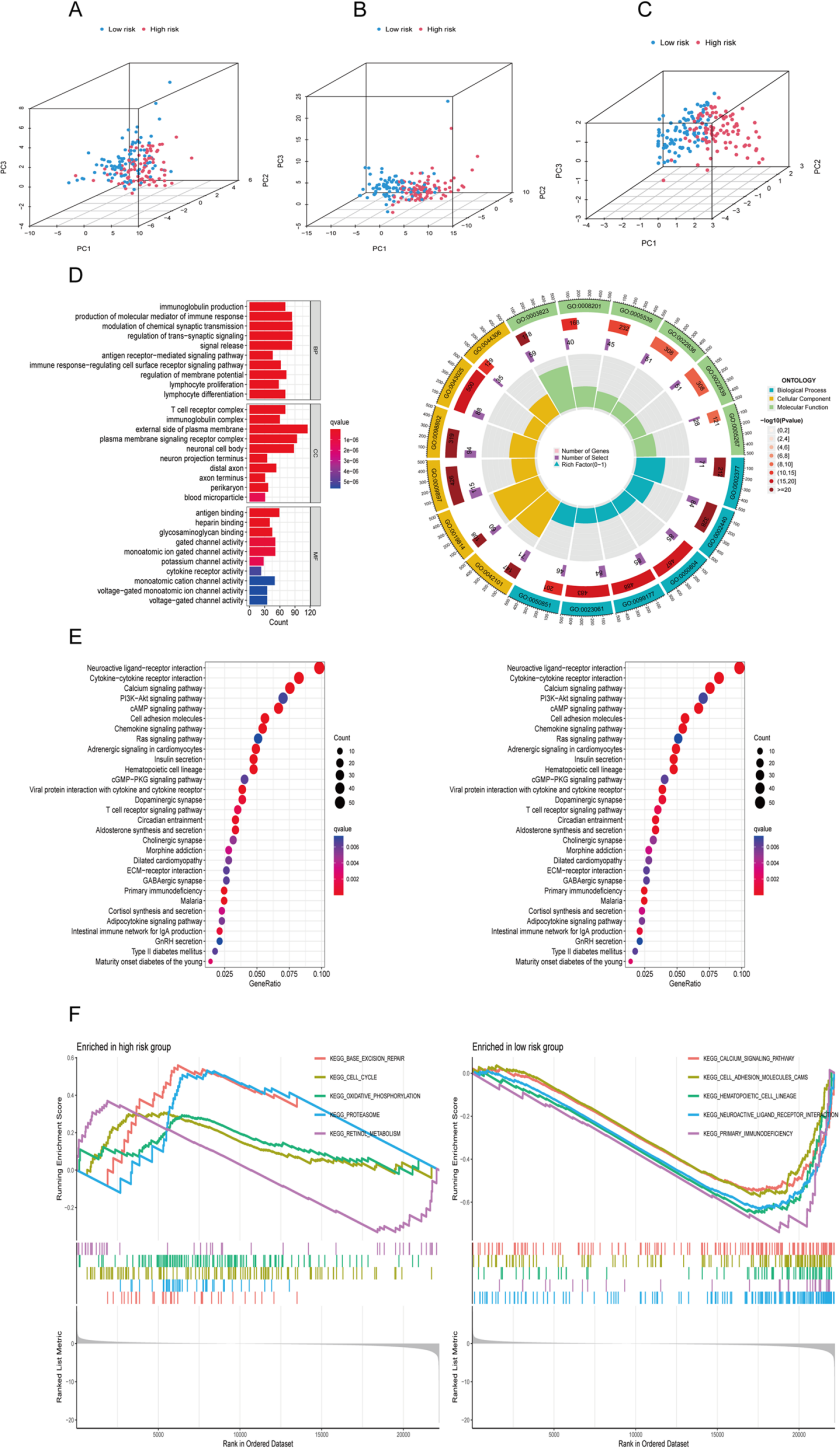
Biological functional and pathway enrichment analysis of the DRLs prognostic signature. **A** PCA about DRGs of patients in two risk groups. **B** PCA about DRLs of patients in two risk groups. **C** PCA about the three DRLs used in the predictive model of patients in two risk groups. **D** GO analysis reveals the diversity of BP, CC, and MF. **E** KEGG analysis shows the significantly enriched pathways. **F** GSEA analysis demonstrates the enriched pathways in two risk groups

PCA was used to visualize the difference in distribution between high- and low- risk groups. The results showed no significant difference in the expression patterns of DRGs, and DRLs between the two risk groups (Fig. 6A-B). However, the 3-DRLs used in the predictive model exhibited the highest discriminatory power in distinguishing between high- and low-risk patients (Fig. 6C).

To investigate the biological functions of DRGs, we performed GO and KEGG enrichment. In terms of biological process (BP), DEGs play important roles in “modulation of chemical synaptic transmission”, “regulation of trans-synaptic signaling”, “signal release” and “production of molecular mediator of immune response”. In the field of cellular components (CC), “external side of plasma membrane”, “plasma membrane signaling receptor complex” and “neuronal cell body” are significantly enriched. In addition, molecular function (MF) analysis showed that deg was significantly enriched in “antigen binding”, “gated channel activity” and “monoatomic ion gated channel activity” (Fig. 6D). These findings suggest that DEGs were involved in metabolism-related biological functions. The KEGG results were consistent with GO analysis, showing significant enrichment in “Neuroactive ligand-receptor interaction”, “Cytokine-cytokine receptor interaction”, “Calcium signaling pathway”, “cAMP signaling pathway”, and “Cell adhesion molecules” (Fig. 6E). In addition, by GSEA analysis, the pathways associated with poor tumor prognosis in the high-risk group were found to be related to “Base excision repair”, “Cell cycle”, “Oxidative phosphorylation”, “Proteasome”, and “Retinol metabolism” (Fig. 6F).

### Analysis of TME characteristics and immune infiltration

**Fig. 7:**
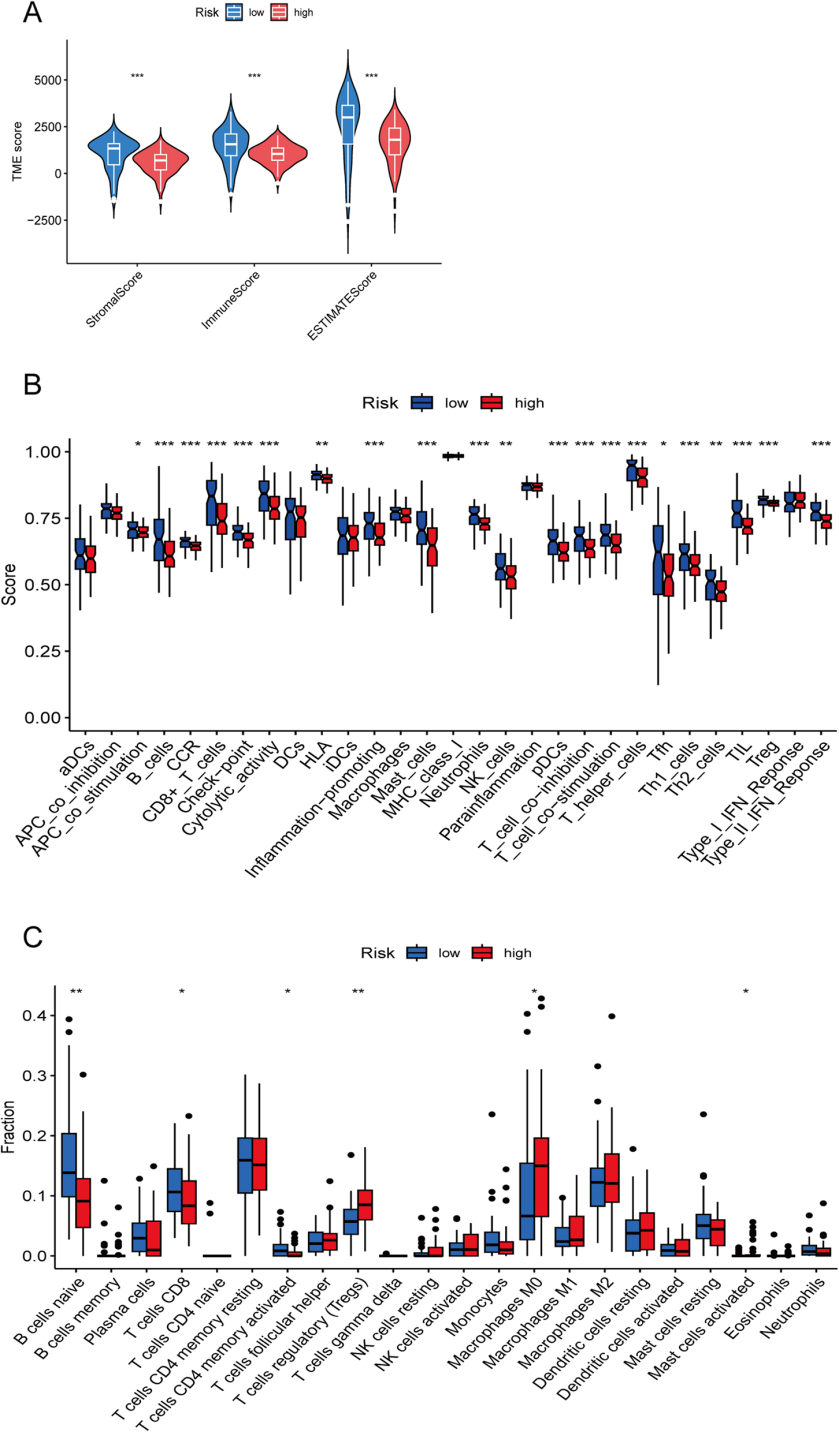
Analysis of immune cell infiltration in PC patients. **A** Differential expression of tumor microenvironment scores (Stromal Score, Immune Score, and ESTIMATE Score) between two risk groups. **B** Infiltration abundance of tumor immune cells in two risk groups. **C** Differential expression of immune functions scores between two risk groups. *, *p* < 0.05; **, *p* < 0.01; ***, *p* < 0.001

In order to clarify the characteristics of the two disulfidptosis subtypes of TME, the immunity score and gene expression between the two groups were calculated in this study. Consist with previous observations, immune score, stromal score and ESTIMATE score were lower in the high-risk group than in the low-risk group (Fig. 7A). We analyzed the proportion of immune infiltration between different PC risk groups and found that regulatory T cells and macrophages infiltration were more abundant in the high-risk group while naive B cells and CD8 T cells were more abundant in the low-risk group (Fig. 7B). We quantified the enrichment scores of different immune cell subgroups to investigate the correlation between risk score and immune functions. The scores of immune-related molecules such as Checkpoint, CCR, and Inflammation-promoting molecules were significantly decreased in the high-risk group compared to the low-risk group (Fig. 7C).

### Analysis of TMB characteristic and drug sensitivity

**Fig. 8:**
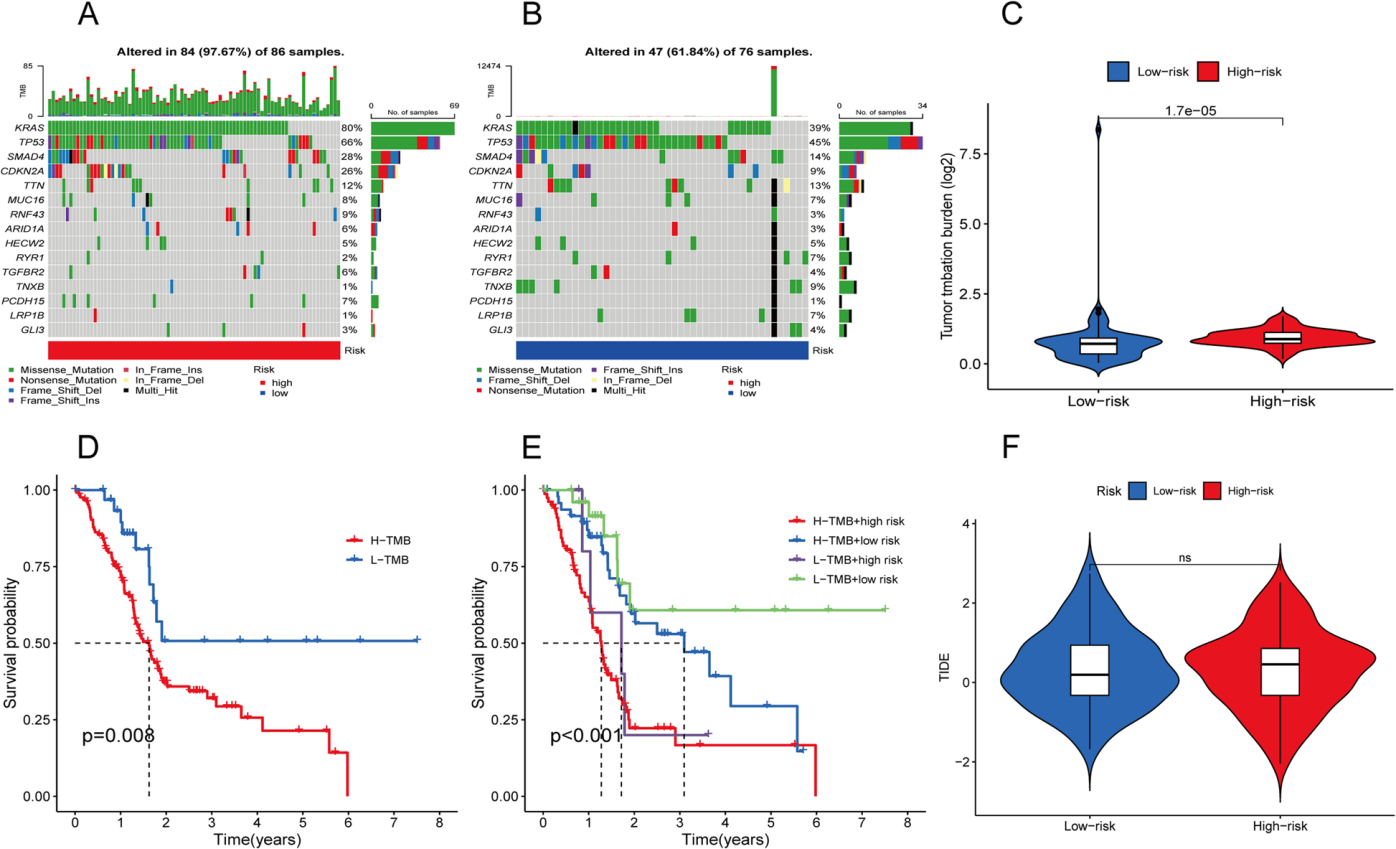
Mutation profile and drug sensitivity analysis of the high- and low-risk groups. **A**-**B** Waterfall plots of somatic mutations in tumors in two risk groups. **C** Analysis of the difference for TMB between two risk groups. **D** The K—M survival analysis of PC patients between high- and low-TMB groups. **E** The K-M survival analysis of PC patients regarding TMB combined with risk score. **F** The violin plot of TIDE analysis for two risk groups

To investigate the differences in cancer-related gene mutations between two risk groups, we obtained somatic mutation data from the TCGA database. As shown in Fig. 8A-B, the examination identified fifteen genes with the highest mutation frequencies, among which the KRAS, TP53, CDKN2A, and SMAD4 genes had high mutation frequencies in both risk groups. Totally, there was significant difference in TMB between the two groups (Fig. 8C). Moreover, subgroup analysis combining TMB and risk scores was effective in predicting patient prognosis. Compared with the other groups, the high TMB and high-risk groups had the worst prognosis, whereas the low TMB and low-risk groups had the longest survival time (Fig. 8D-E).

**Fig. 9:**
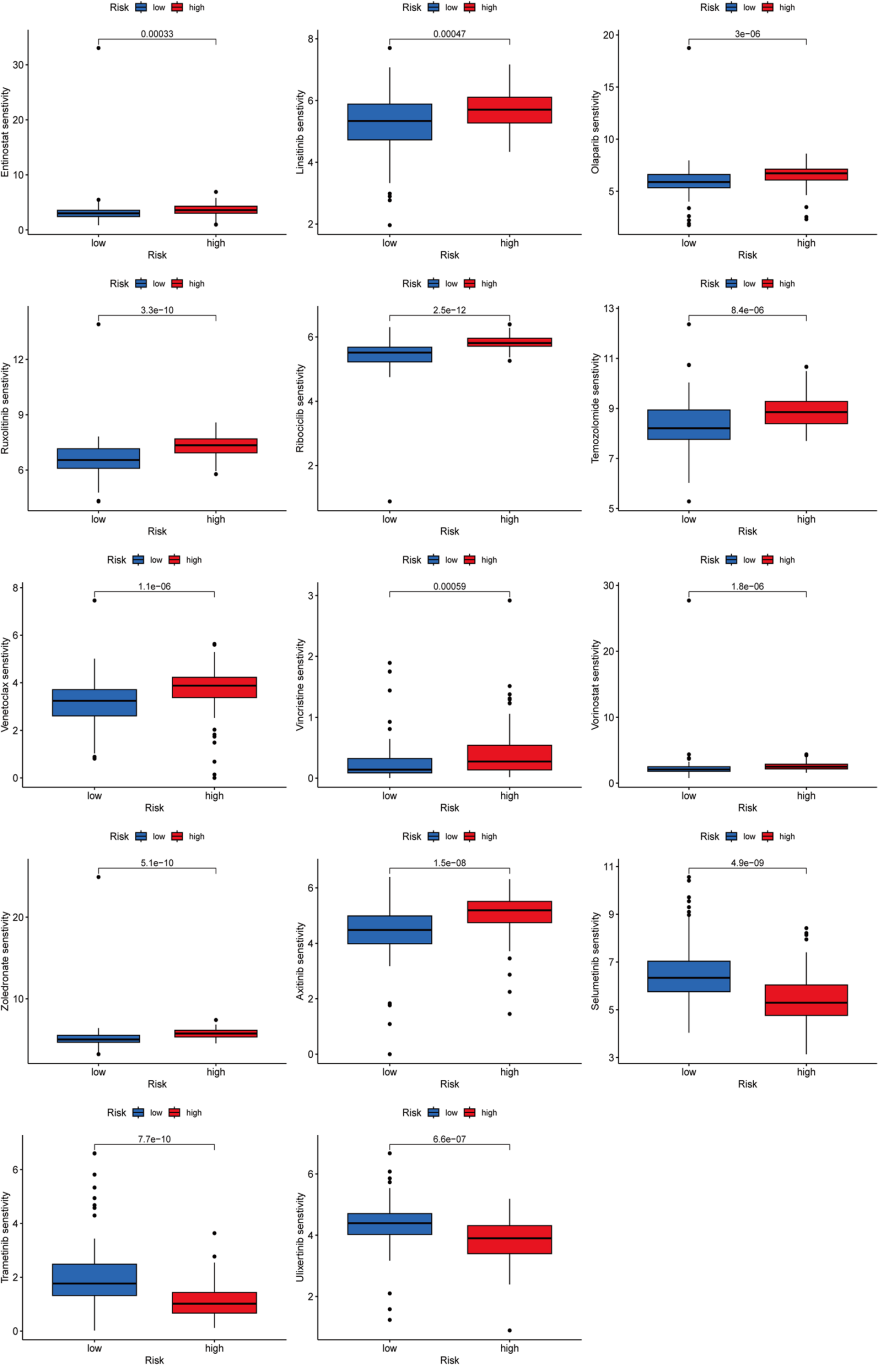
The relationship between risk groups and drug sensitivity

Immune checkpoint blockade (ICB) has made significant progress in cancer treatment [21, 22]. However, ICB therapy is effective in only a subset of patients. To further explore the role of risk scores in immunotherapy, we applied the TIDE score to assess potential immune dysfunction in tumors and regional lymph nodes. The results showed that patients in the two risk groups have no significant probability of responding to immunotherapy (Fig. 8F). We also analyzed the sensitivity of chemotherapy drugs between two risk groups. Several chemotherapy drugs were widely used in clinical, rendering Entinostat, Linsitinib, Olaparib, Ribociclib, Ruxolitinib, Temozolomide, Venetoclax, Vincristine, Vorinostat, and Zoledronate were more suitable for patients in high-risk category (Fig. 9). Conversely, Axitinib, Selumetinib, Trametinib and Ulixertinib were indicated their higher sensitivity to patients classified as low-risk (Fig. 9).

## Discussion

PC, is a malignant tumor with a poor prognosis, and it is a major challenge to improve its overall survival rate. In recent years, the incidence of pancreatic cancer has been on the rise globally, especially among young people. Although screening is an important method of detecting patients with early-stage PC, it is not recommended for the general population due to the low incidence of PC and the small benefit of screening. In addition, the accuracy of existing screening methods is not satisfactory and some of them may have negative effects on human health, such as pain and anesthesia-related adverse reactions after endoscopic ultrasonography (EUS) examination, acute pancreatitis, and even hospitalization after endoscopic retrograde cholangiopancreatography (ERCP), as well as anxiety and psychological effects [23].

Many new forms of RCD have attracted great attention, such as ferroptosis, cuproptosis, autophagy-dependent cell death, lysosome-dependent cell death, endogenous cell death and reticulocyte death, all of which are important for TME therapy [24]. Ferroptosis has the ability to activate tumor immune cells by transmitting chemotactic signals, and ferroptosis inducers play a role in suppressing tumor immunotherapy [25]. Previous literature suggested that gemcitabine and cisplatin combination therapy induced ferroptosis in a GSH-independent manner in pancreatic ductal adenocarcinoma [26]. Moreover, ferroptosis was shown to be associated with sensitivity to gemcitabine in PC [27–29]. A recent study showed that high expression of SLC7A11 accelerates intracytoplasmic NADPH depletion, especially under glucose starvation, which may inhibit ferroptosis and induce a new form of cell death, namely disulfidptosis [4]. However, because of limited studies on the application of disulfidptosis in PC, further studies are necessary to explore its potential in cancer therapy.

Personalized medicine is increasingly utilizing biomarkers such as lncRNAs, which provide more accurate diagnosis, prognosis, and treatment options [30]. LncRNAs extensively regulate the biological behaviors of PC, such as promoting tumor angiogenesis, metastasis, proliferation, immune escape, and metabolic reprogramming [31–35]. In this study, we generated a DRL signature to predict the prognosis and immune microenvironmental status of PC patients. The results showed that the risk score of this model was an independent predictor of PC patients. Combined with the evaluation of ROC and nomogram, it suggested that the constructed DRLs signature can accurately predict the prognosis of PC patients. Among the three DRLs used for characterization, AP005233.2 was found to be associated with metabolism and patient prognosis in intrahepatic cholangiocarcinoma [36], lung adenocarcinoma [21] and PC [22]. Wang et al. demonstrated FAM83A-AS1 inhibited Hippo pathway activation to active YAP to promote the proliferation and EMT of pancreatic cancer cells. Besides, Hippo pathway play an important role in regulating cell proliferation, regeneration and organ size control [37]. FAM83A-AS1 was also a necroptosis-related lncRNA regulated malignancy and glycolysis in lung adenocarcinoma [38, 39]. TRAF3IP2 − AS1, was related to ferroptosis [40], pyroptosis [41] and N6-methyladenosine (m6A) [42] and has utility in predicting PC prognosis, depicting the tumor immune microenvironment and guiding immunotherapy. TRAF3IP2-AS1 is a hub m6A-lncRNA with a dysregulated expression pattern in the panel, which can inhibit the proliferation of PC tumors in vitro and in vivo. Xu et al. found that knocking out TRAF3IP2-AS1 reduced cell apoptosis and altered cell cycle distribution. After gene knockout, the activity of caspase 3 and caspase 9 was significantly reduced, indicating that TRAF3IP2-AS1 may affect PC cell apoptosis through the mitochondrial pathway [42].

Based on these three DRLs, we developed a new clinical prognostic model that is more suitable for clinical application than some of the signatures already identified. In our study, we randomized the PC cohort into training and testing subsets. Subsequently, patients were categorized into high- and low-risk groups based on their respective risk scores calculated using the developed model. In terms of survival curves, the high-risk group had a worse prognosis which was consistent with the clinical subgroup analysis (except for M1 and G3-4). The ability of risk scores to predict the prognosis of PC patients was more prominent than traditional TNM staging and other clinicopathologic features, as confirmed by ROC curves, C-index, and K-M survival curves. In addition, a nomination graph containing clinical variables and risk scores showed that compared to existing clinical staging systems, risk scores have stronger predictive power. The risk score is not related to important prognostic factors for PC, and we used median values to divide patients into different groups. Using median for classification is considered a more practical and objective method, especially compared to the optimal cutoff values, which may only perform well in specific populations and lack universality.

To further understand their biological properties, we performed GO and KEGG analysis. GO analysis showed that DRLs were mainly associated with cellular signaling, suggesting that they are closely associated with cellular metabolism, which is consistent with the fact that NADPH depletion and disulfide stress leading to the disulfide bond formation in protein molecules triggering disulfidptosis, for both are closely related to energy supply and cellular metabolism [43]. KEGG analysis showed that “Neuroactive ligand − receptor interaction”, “Cytokine − cytokine receptor interaction”, and “Calcium signaling pathway” were significantly enriched. Although neuroactive ligand-receptor interactions are primarily associated with neurological disorders, there is growing evidence that they are involved in cancer progression and metabolism. It has been shown that in PC, perineural invasion-triggered cholinergic signaling favors tumor growth by promoting an immune-suppressive microenvironment characterized by impaired CD8 T-cell infiltration and a reduced Th1/Th2 ratio [44]. Mechanisms by which Ca^2+^ channels act on tumors are complex and can affect tumor progression in several ways. Ca^2+^ has been reported to activate the NF-κB, NFAT and CREB pathways, thereby playing an important role in tumor immune cells and progression [45, 46]. In addition, according to GSEA analysis, the pathways associated with poor tumor prognosis were significantly enriched in the high-risk group, including “Base excision repair”, “Cell cycle”, “Oxidative phosphorylation”, “Proteasome”, and “Retinol metabolism” in terms of pathway.

Recent studies have shown that disulfidptosis is strongly associated with immune infiltration, with high disulfiram subtypes exhibiting higher immune scores [4]. The results indicate that patients with lower risk scores have more active TIME and more immune cell infiltration, which may be beneficial for immunotherapy [47]. Our findings were consistent with the previous view that a high degree of CD8 T cells and naïve B cells infiltration usually implies a better survival prognosis [48]. Similar to our model, three recent prognostic models in PC showed that low levels of CD8 T cells were associated with poor prognosis [49–51]. In addition, there was a significant reduction of Th1 cells as key cells that generate a durable anti-tumor immune response in the high-risk group [52], which may contribute to the poorer prognosis of this group. The tumor immune environment is a complex environment, and in addition to immune cells, various factors such as immune checkpoints, regulatory cells, inflammatory factors, and tumor microenvironment can influence the immune function. Although little is known about the immune regulatory function of Treg cells in such tumors, their presence in the tumor matrix is associated with T cell-mediated immune response suppression and impaired immune surveillance [53]. The increase of Treg cells in tumors is also related to blocking the recruitment of CD8 + T cells and inhibiting the immunogenic function of antigen-presenting cells [54, 55]. Pancreatic TME, especially infiltrating inflammatory cells (mainly macrophages), is an important contributing factor to PC aggressiveness and resistance to treatment [56]. Macrophages in TME are often referred to as tumor-associated macrophages and contain three phenotypes. Among them, M0 macrophages, as a non-polarized subtype, aere an independent predictor of poor prognosis in PC patients [57, 58]. Tekin et al. discovered that M0 macrophages secreted matrix metalloprotease 9 (MMP9) which induces mesenchymal transition in PC cells [59]. Although it is still unclear whether M0 macrophages promote tumor growth by directly contacting tumor cells or by inhibiting T cell function, Xu et al. show that M0 macrophages can promote the growth of pancreatic cancer in vivo experiments [60]. Interestingly, we found that patients with low-risk scores simultaneously had higher stromal scores, immune scores, and ESTIMATE scores. Although the low-risk scoring group has more abundant infiltrating immune cells, a higher matrix score may indicate that infiltrating immune cells are more likely to be blocked by matrix components, such as extracellular matrix secreted by cancer fibroblasts [61, 62]. The infiltration of these immune cells into tumor tissue and their anti-tumor efficacy may be weakened [61].

Immunotherapy is becoming a prominent trend in anti-tumor treatment for various types of cancer, divided into the following main categories: immune checkpoint inhibitions (ICIs), Tumor vaccines, chimeric antigen receptor T cells (CAR-T), and non-specific immunotherapy [63, 64]. ICIs play a crucial role in maintaining appropriate immune responses and protecting healthy tissues from immune attacks under normal physiological conditions [65]. This involves regulating the recognition of antigens by T cell receptors through co stimulation or inhibition of signal transduction [66]. ICIs therapy has shown encouraging progress in many malignant tumors and chemotherapy resistant cancers, as it has natural immunogenicity by infiltrating T cells into the TME and promoting cytotoxic signaling pathways [67]. According to reports, TIDE is an accurate method for predicting cancer patients’ response to ICI treatment [68]. Unfortunately, however, according to the TIDE results, the probability of immune escape was not significantly different between the two risk groups, which may explain why single-agent programmed death 1 ligand (PD-L1) or cytotoxic T-lymphocyte-associated protein 4 (CTLA-4) inhibitors is ineffective for PC [69–71].

Duplicate somatic mutations in specific genes have been identified as potential cancer initiators [72, 73], among which, KRAS, CDKN2A, SMAD4, and TP53 are frequently mutated in PC [74]. Previous studies have shown that KRAS mutations first lead to pancreatic precancerous lesions, followed by inactivation of CDKN2A, TP53, and SMAD4 [75, 76]. Recent studies have shown that inactivation of SMAD4, KRAS and TP53 genes can promote cellular aerobic glycolysis and tumor invasiveness [77–80]. The number. High score of somatic mutations presenting in the tumor genome indicated by TMB is associated with poor prognosis in PC patients [81], which is consistent with our study. In recent years, the discovery of anti-tumor targets has led to the development of cancer therapy from traditional cytotoxic drugs to new specific anti-tumor drugs [82, 83]. Our pharmacosensitivity analysis showed that high-risk PC patients may be more sensitive to Entinostat, Linsitinib, Olaparib, Ribociclib, Ruxolitinib, Temozolomide, Venetoclax, Vincristine, Vorinostat, and Zoledronate. Olaparib is widely used in patients with a germline BRCA mutation and metastatic pancreatic cancer [84, 85]. It is worth noting that Linsitinib, IGF-1R inhibitors is exploited for therapeutic benefit as effective adjuvant anticancer treatments for PC patients with deacetylated ENO2 [86].

The aim of this study was to investigate the possible association between DRLs and patient prognosis constructing a novel and innovative model. The resulting model was found to have good predictive forecasting potential through multi-perspective exploration and validation. However, despite the good performance of the model in both cohorts, it still has some limitations. Firstly, the data were obtained from a single database, and therefore, there may be data bias. Due to limited research on the three selected lncRNAs, we are unable to obtain comprehensive lncRNA annotations and clinical information from databases such as International Cancer Genome Consortium (ICGC). This limitation highlights the continued importance of lncRNA, which is still limited to some extent by current technology. Secondly, the model needs to be validated using prospective multicenter studies with larger sample sizes, and complete follow-up information are necessary to further validate and elucidate the mechanism of action of DRLs in PC.

## Conclusions

This paper systematically analyzed the role of DRLs in pancreatic carcinoma prognosis and developed a prognostic model using the relationship between TMB, TME and clinical features. In addition, the validity of DRLs markers as markers of possible treatment response was evaluated. Taken together, these findings reveal the clinical importance of DRLs and provide a foundation for future research.

## Supplementary Information

Supplementary Material 1: Fig. 1. The nomogram to predict the 1-, 3-, and 5-year overall survival (OS) rate of PC patients (excluding stage III patients).

Supplementary Material 2: Fig. 2. The calibration curve for evaluating the accuracy of the nomogram model in 1-, 3-, and 5-year categories (excluding stage III patients).

## Data Availability

All the data are available in TCGA (https://portal.gdc.cancer.gov/), which are public functional genomics data repositories.
