# Supplementary figures and images for: Disulfidptosis classification of pancreatic carcinoma reveals correlation with clinical prognosis and immune profile
Source: Hereditas. 2025 Feb 22;162:26. doi: 10.1186/s41065-025-00381-z (PMC11846472; doi:10.1186/s41065-025-00381-z)

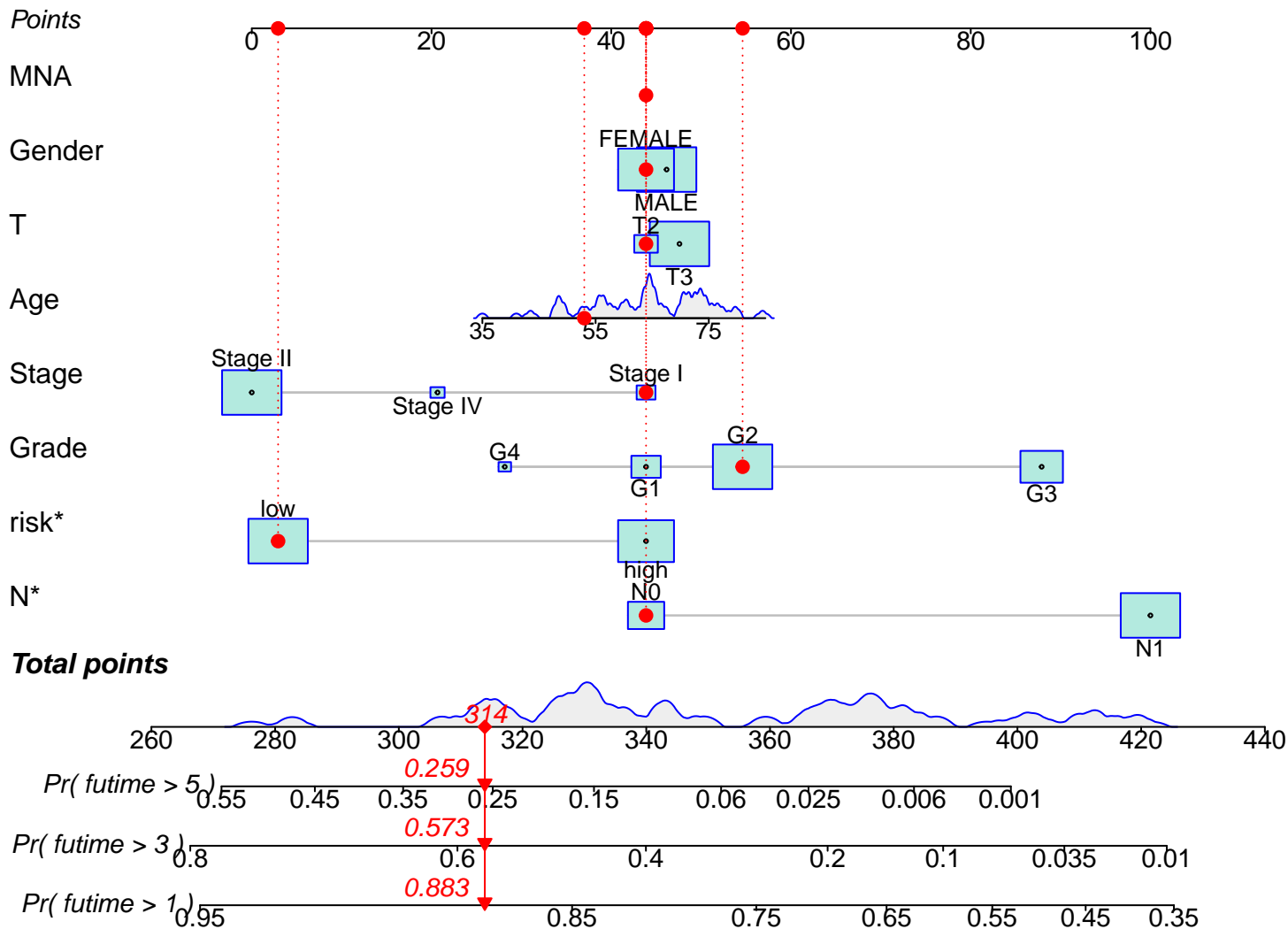

Supplement: Supplementary file 1 — Supplementary Material 1: Fig. 1. The nomogram to predict the 1-, 3-, and 5-year overall survival (OS) rate of PC patients (excluding stage III patients). [file 41065_2025_381_MOESM1_ESM.pdf]

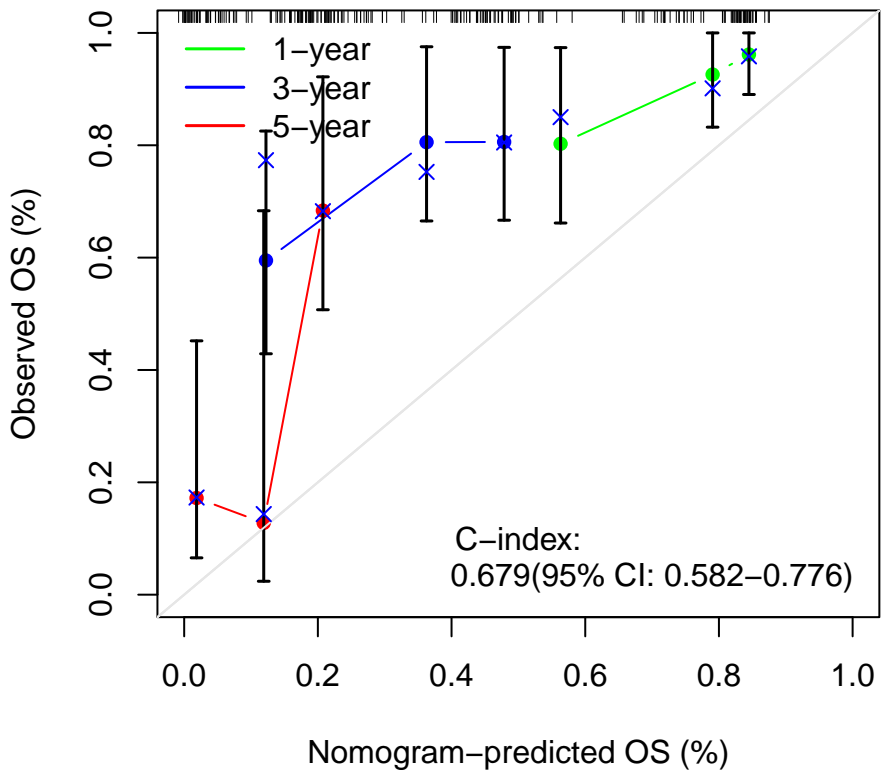

Supplement: Supplementary file 2 — Supplementary Material 2: Fig. 2. The calibration curve for evaluating the accuracy of the nomogram model in 1-, 3-, and 5-year categories (excluding stage III patients). [file 41065_2025_381_MOESM2_ESM.pdf]
